# David J. Glass elected to the U.S. National Academy of Sciences

**DOI:** 10.1186/s13395-024-00343-5

**Published:** 2024-07-09

**Authors:** Se-Jin Lee, Bruce Spiegelman, Kevin Campbell

**Affiliations:** 1 The Jackson Laboratory, University of Connecticut School of Medicine, Farmington, USA; 2 Dana Farber Cancer Institute, Harvard Medical School , Boston, USA; 3 University of Iowa Carver College of Medicine, Howard Hughes Medical Institute, Iowa City, USA

On April 30, 2024, David J. Glass was elected to the U.S. National Academy of Sciences in recognition of his exceptional contributions to our understanding of the control of skeletal muscle homeostasis. David obtained his M.D. from New York Medical College and then decided to forego any further medical training and instead pursue a full-time career in biomedical research. After carrying out postdoctoral training with Charles Cantor and then Stephen Goff, both at Columbia University, David decided to leave academia to join the biotechnology company, Regeneron. It was at Regeneron that David began his studies on skeletal muscle biology, which has remained the primary focus of interest throughout his career.

Over a span of several years at Regeneron, David firmly established himself as a major force in the skeletal muscle field with a series of high-profile papers reporting the discovery and characterization of molecules and signaling pathways that are critical to the regulation of skeletal muscle hypertrophy and atrophy. With respect to muscle hypertrophy, working in collaboration with George Yancopoulos at Regeneron, David published a paper in *Science* in 1999 showing that two intracellular pathways, PI3-Akt and Raf-MEK-ERK, oppose one another in regulating muscle fiber growth, with Akt acting to promote hypertrophy [1]. They quickly followed this up with two *Nature Cell Biology* papers in 2001 showing that Akt activation is what mediates the hypertrophic response of muscle to insulin-like growth factor-1 [2, 3]. Although we tend to take for granted the role of Akt in mediating skeletal muscle growth, it is important to keep in mind that at the time, their findings challenged the prevailing thought that IGF-1 may act through calcineurin to regulate hypertrophy.

In terms of muscle atrophy, David published a paper in *Science* that same year reporting the identification of two muscle-specific E3 ubiquitin ligases, MuRF1 and MAFbx, that were upregulated in several different models of muscle atrophy. They further showed that when these were knocked out in mice, there was sparing of muscle following denervation [4]. MAFbx was independently discovered by the late Alfred Goldberg and named atrogin-1 [5]. This *Science* paper remains David’s most highly cited paper, with over 3500 citations, and numerous follow-up studies by many investigators, including David, have shown that these ubiquitin ligases are upregulated in virtually every catabolic state that has been examined. Targeting these ubiquitin ligases can mitigate muscle atrophy due to a wide range of physiologic causes. David’s lab also discovered that the primary substrate of MuRF1 in mediating atrophy is a major constituent of the sarcomere, namely myosin heavy chain [6]. David further showed that these hypertrophy and atrophy pathways are linked through the transcription factor FOXO, with Akt signaling being capable of preventing muscle atrophy by blocking the ability of FOXO to induce expression of these ubiquitin ligases [7].

After spending 15 years at Regeneron, David moved to the Novartis Institutes for BioMedical Research, Inc. to lead their musculoskeletal program. At Novartis, he became interested in understanding the roles of the TGF-ß-related ligands, myostatin, GDF‐11, and activin A, in regulating skeletal muscle. In addition to carrying out important basic research elucidating fundamental mechanisms by which these ligands act to regulate muscle growth and metabolism, David led the development of a monoclonal antibody (bimagrumab) directed against myostatin/activin receptors that was taken into numerous clinical trials for a wide range of muscle indications [8]. Among these trials, perhaps the most noteworthy was a phase 2 trial in which Novartis showed striking beneficial effects in reducing total body fat and improving glucose metabolism in obese patients with type 2 diabetes [9]. The results from this trial have been a major driver of the recent surge in interest in the use of myostatin/activin A inhibitors for obesity.

In 2019, David returned to Regeneron to become Vice President of Aging/Age-Related Disorders. In this regard, one of the remarkable aspects of David’s work is that he has made such fundamental contributions elucidating basic mechanisms underlying muscle hypertrophy and atrophy while working entirely within the research mission of biotechnology and pharmaceutical companies. Moreover, in addition to his scientific contributions, David has had a major impact on the skeletal muscle field in many other ways, including organizing multiple major conferences and, along with Michael Rudnicki and one of us (KC), co-founding this journal dedicated to skeletal muscle biology. Although David spent most of his career in biotech, he has also made many contributions to academia and is currently Senior Lecturer of Cell Biology at Harvard Medical School. We hope that you will join us in thanking David for all of his contributions to our field and in congratulating him for his well-deserved election to the National Academy of Sciences.
